# Examining the Effect of an Anion-Binding Reagent on the Structure of Deprotonated Leucine Enkephalin Using Cryogenic-Ion Infrared Action Spectroscopy

**DOI:** 10.1021/acs.jpca.5c03984

**Published:** 2025-08-28

**Authors:** Madeline Schultz, Nwanne D. Banor, Katja Ober, America Y. Torres-Boy, Maleesha T. Fernando, Miyuru M. Wellalage, Neil A. Ellis, Gert von Helden, Daniel A. Thomas

**Affiliations:** † Department of Chemistry, 4260 University of Rhode Island, Kingston, Rhode Island 02881, United States; ‡ Fritz-Haber-Institut der Max-Planck Gesellschaft, Faradayweg 4-6, Berlin 14195, Germany

## Abstract

Biomolecular systems feature a complex interaction network comprising numerous intra- and intermolecular interactions. By isolating biomolecules under vacuum conditions, the intricate balance between specific interaction motifs can be characterized with precise control over conditions. In this study, we apply cryogenic-ion infrared action spectroscopy and electronic structure methods to examine the structural changes in the deprotonated form of the model peptide leucine enkephalin (YGGFL) upon complexation with diserinol isophthalamide (DIP), an anion-binding reagent. The low-energy conformer of the uncomplexed, deprotonated peptide ([YGGFL - H]^−^) adopts a noncanonical turn structure stabilized by intramolecular ionic hydrogen bonding to the C-terminal carboxylate moiety. Despite the favorability of DIP to strongly coordinate with carboxylate residues, we find that the structure of the peptide is largely unaffected by the binding of DIP. Instead, DIP only partially coordinates with the carboxylate moiety and is positioned below the backbone turn of YGGFL to engage in additional hydrogen bonding interactions. These findings underscore the stability of the turn structure and the strong energetic penalty imposed by disruption of this motif even when strong intermolecular coordination is expected.

## Graphical abstract

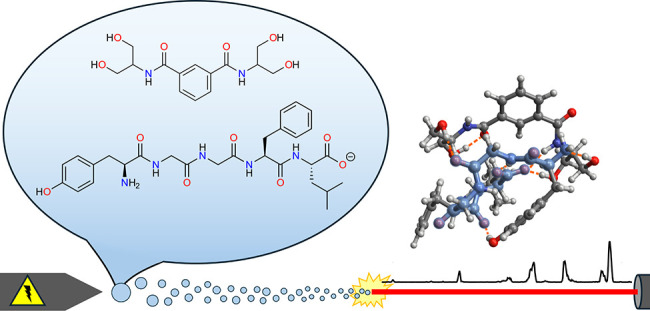

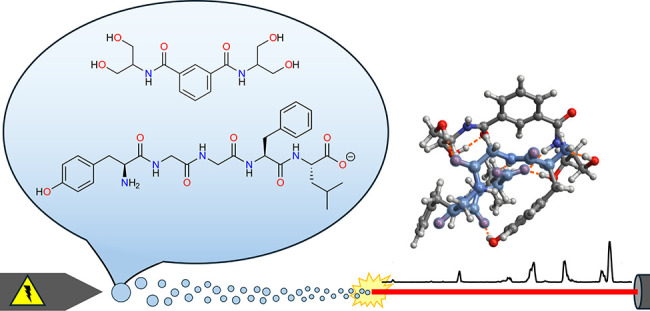

## Introduction

Biomolecular secondary, tertiary, and quaternary structures are stabilized by a network of inter- and intramolecular interactions. The subtle balance between these structural motifs governs system behavior across protein–water,^1–4^ protein–protein,^5–7^ and protein–ligand interactions.^8–12^ A precise description of these interactions is especially relevant for elucidating the structure and evolution of highly dynamic systems, such as intrinsically disordered proteins and biomolecules undergoing liquid–liquid phase separation.^9–15^ However, the inherent complexity of these interactions in the condensed phase presents a significant experimental challenge.^3,9^ Mass spectrometry (MS) and related techniques have emerged as a powerful tool for the analysis of biological structure that complements traditional condensed-phase studies.^16–20^ Prominent techniques include hydrogen–deuterium exchange (HDX) MS for the comparative analysis of structure and dynamics,^16,21–23^ ultraviolet photodissociation (UVPD) or electron-based dissociation (ExD) for the identification of structure and noncovalent interactions,^17,24–30^ ion mobility spectrometry (IMS) for the elucidation of three-dimensional structure,^19,31–35^ and action spectroscopy for the systematic analysis of isolated conformers.^20,36–39^ For small biological molecules such as peptides, infrared action spectroscopy can be leveraged to reveal the noncovalent interaction network and analyze the contribution of each interaction to higher-order structure.^37,40–42^

This work is concerned with changes in the peptide conformational landscape upon binding of a small molecule to charged sites. By contrast between bound and unbound conformations, these systems reveal the structural stabilization provided by intramolecular ionic hydrogen bonding. Previously, the model pentapeptide leucine enkephalin (YGGFL) has served as a test case for such studies. The unbound, protonated form of YGGFL has been widely characterized by structure-sensitive techniques including infrared multiple-photon dissociation (IRMPD),^43^ ultraviolet photodissociation (UVPD),^44^ ion mobility spectrometry (IMS),^34,45^ and infrared-ultraviolet (IR-UV) double resonance photofragment spectroscopy.^41^ It is known to adopt a type II′ β-turn with prominent ionic hydrogen bonds to the protonated N-terminus.^46,47^ To investigate the contribution of charge-site interactions to the stability of the turn structure, the complex between protonated YGGFL and 18-crown-6 was previously examined by cryogenic-ion infrared (IR) action spectroscopy.^48^ 18-crown-6 is known to bind monoalkylammonium residues via intermolecular hydrogen bonding,^48–58^ thereby disrupting the intramolecular interactions between the N-terminus and the backbone carbonyl groups. Complexation was observed to result in reorientation of the protonated N-terminus, but surprisingly, the overall β-turn structure of the peptide was preserved.^48^

Herein, we examine the contribution of ionic hydrogen bonding to the structure of deprotonated YGGFL^59^ by complexing the peptide with an anion-binding reagent, diserinol isophthalamide^60^ (DIP, Scheme 1), which binds carboxylate moieties of biomolecular ions during the electrospray process.^60,61^ Similar to studies on the protonated form of YGGFL, structural characterization of both the bound and unbound conformers provides information on the importance of intramolecular interactions of the carboxylate residue. The uncomplexed, deprotonated form of YGGFL has previously been characterized by IR action spectroscopy and IMS.^62^ As with protonated YGGFL, the low-energy conformer of the deprotonated molecule adopts a turn structure with multiple ionic hydrogen bonds to the charge site, in this case the C-terminal carboxylate group. However, the bond angles indicate a noncanonical turn structure rather than the type II′ β-turn observed for the protonated form. In this work, we investigate whether this turn structure is preserved upon complexation of the peptide with DIP, which is expected to compete for ionic hydrogen bond interactions with the carboxylate moiety.

**Scheme 1:**
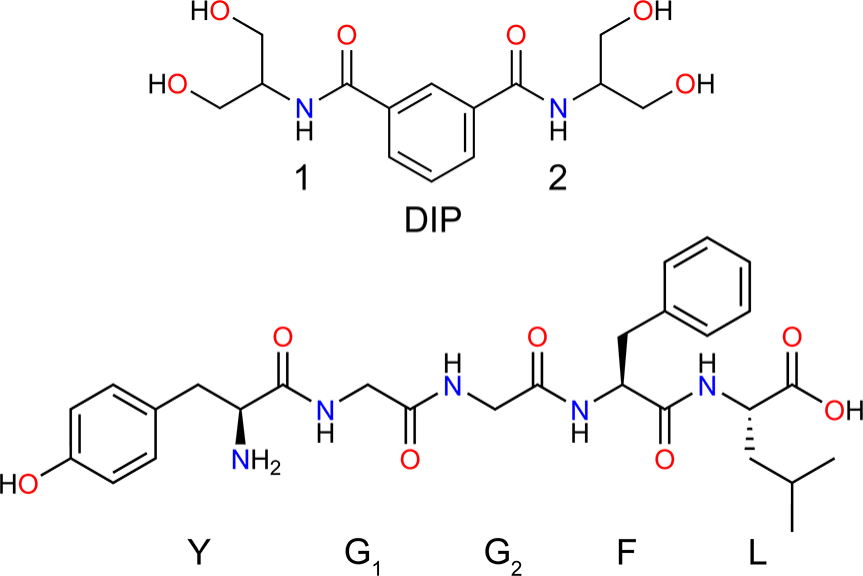
Structure of Diserinol Isophthalamide (DIP) and Leucine Enkephalin (YGGFL)

To characterize the structure of deprotonated YGGFL and its complex with DIP, ions are prepared by electrospray ionization mass spectrometry (ESI–MS) and captured in helium nanodroplets for IR action spectroscopy. This approach has been applied extensively for the characterization of biomolecular ion structure.^48,63–68^ Nanodroplet-entrained ions are cooled to 0.4 K, resulting in population of only low-energy states and enabling acquisition of highly resolved IR spectra. Ion photon absorption results in evaporation of He atoms and ultimately generation of the bare ion. Monitoring ion yield as a function of photon wavelength yields an IR action spectrum. The experimental spectra are compared to predicted conformer spectra obtained from electronic structure methods to identify the peptide structure.

## Methods

### Experimental Methods

Infrared action spectroscopy of ions in helium nanodroplets was performed using custom instrumentation described previously (Figure S1).^67–69^ Briefly, ions were generated by nanoelectrospray ionization (nESI) from Pd/Pt-coated pulled-glass needles prepared in-house. Ions were mass-to-charge (*m*/*z*) selected by a quadrupole mass filter, bent 90° by a quadrupole deflector, and loaded into a hexapole ion trap held at ca. 90 K. A beam of helium nanodroplets, generated by a pulsed Even-Lavie valve^70^ at a pressure of ca. 70 bar and a temperature of 21 K,^68^ traversed the ion trap, resulting in ion capture. Ion-doped droplets escaped the trap and traveled to a ring-electrode ion guide, wherein they were irradiated with IR photons generated by the free-electron laser (FEL) at the Fritz Haber Institute.^71^ FEL pulses were ca. 10 μs in length and composed of ca. 10^4^ ps-length micropulses at a repetition rate of 1 GHz.^67^ Energy from the absorption of resonant photons was dissipated via intramolecular and intermolecular vibrational redistribution, ultimately causing He evaporation. The successive absorption of photons from individual FEL micropulses resulted in nanodroplet evaporation and the generation of bare ions,^72^ which were detected by an off-axis time-of-flight (TOF) detector.

The intensity at a given photon energy was obtained as the integrated target *m*/*z* ion signal from the average of 25 FEL macropulses. Measurements were collected between 1250 and 1750 cm^–1^ in 1 cm^–1^ steps, and the ion trap was refilled between each measurement step. Laser power was adjusted depending on line intensity to achieve operation in a pseudolinear response regime,^72^ and combined spectra were scaled by the intensity of lines overlapping between scans. Intensities were corrected for photon fluence by scaling with a factor of (laser power × wavenumber)^−1^. Each region was scanned at least twice and averaged to ensure reproducibility.

Sample solutions were prepared from leucine enkephalin salt hydrate (purity ≥ 95%, Merck Millipore, Darmstadt, Germany) and diserinol isophthalamide (DIP), which was synthesized as described previously.^60^ To prepare sample solutions, YGGFL was dissolved in 50/50 H_2_O/MeOH (v/v%), and DIP was dissolved in H_2_O. From 1 mM stock solutions, samples were then diluted in 50/50 H_2_O/MeOH (v/v%). YGGFL was prepared at a final concentration of 150 μM. For study of the complexed species, a solution was prepared at concentrations of 500 μM DIP and 50 μM YGGFL.

### Conformational Sampling via CREST

Conformational sampling was performed for deprotonated YGGFL ([YGGFL - H]^−^), the complex between YGGFL and DIP ([YGGFL + DIP - H]^−^), and the complex between acetate and DIP ([CH_3_OO^–^ + DIP]^−^) using the conformer-rotamer ensemble sampling tool (CREST) developed by Pracht, Grimme, and co-workers, wherein low-energy conformers are identified using metadynamic simulations with semiempirical extended tight-binding (xTB) methods.^73–75^ Sampling was performed at the GFN2-xTB level of theory^76^ with the default energy window of 25 kJ mol^–1^ for [YGGFL - H]^−^ (carboxylate and phenolate deprotomers), [YGGFL + DIP - H]^−^ (carboxylate, phenolate, and DIP alkoxide deprotomers), and [CH_3_OO^–^ + DIP]^−^. An additional search for [YGGFL + DIP - H]^−^ in the carboxylate form was performed using the noncovalent interactions (nci) run-type, which generates an ellipsoidal potential to encapsulate noncovalently bound species during the metadynamic simulations and disfavor dissociation.^77^ The ellipsoidal potential wall was scaled by a factor of 2. Further conformational sampling for [YGGFL + DIP - H]^−^ (carboxylate, phenolate, and alkoxide deprotomers) was performed using the LEDE-CREST variant designed for noncovalent clusters.^78^

### Geometry Optimization and Frequency Calculations via ORCA

For the carboxylate deprotomers, all structures obtained from CREST were optimized within the ORCA software package^79,80^ using the B3LYP hybrid density functional with the D3BJ empirical dispersion correction^81–84^ and the def2-TZVP basis set.^85–87^ Unless otherwise noted, the RIJCOSX approximation^88^ was used with the def2/J auxiliary basis set.^89^ Low-energy conformers were additionally optimized using the long-range parametrized CAM-B3LYP­(D3BJ) hybrid density functional^90^ and ωB97X-D3 hybrid density functional^91^ with the def2-TZVP basis set. Separately, the CAM-B3LYP­(D3BJ)/def2-TZVP level of theory was used for optimization of phenolate and deprotonated DIP conformers obtained from CREST. Finally, conformers obtained from LEDE-CREST were manually screened using the *measure cluster* feature of VMD,^92^ and unique conformers were optimized at the CAM-B3LYP­(D3BJ)/def2-TZVP level of theory.

For the lowest-energy conformer of [YGGFL - H]^−^, B3LYP­(D3BJ) optimization was also performed with the ma-def2-SVP,^85,93^ ma-def2-TZVP,^93^ and def2-TZVPP basis sets.^85^ The auxiliary basis set aug-cc-pVTZ/JK^94^ was used with the minimally augmented basis sets, ma-def2-SVP and ma-def2-TZVP. Additionally, the lowest-energy conformer was optimized with the PBE0 hybrid density functional^95,96^ and D3BJ dispersion correction^86,87^ with the ma-def2-SVP, ma-def2-TZVP, def2-TZVP, and def2-TZVPP basis sets. Harmonic vibrational normal-mode frequencies were calculated for all low-energy structures using analytic second derivatives of the Born–Oppenheimer potential. Frequencies were convoluted with Gaussian distributions with a full width at half-maximum of 0.3% (in wavenumber) centered at each frequency and scaled by best fit to experimental spectra using Pearson and Spearman correlation coefficients,^97^ with scaling factors ranging from 0.96 to 0.99.

## Results and Discussion

### Spectroscopy and Conformational Analysis of Deprotonated YGGFL

Shown in Figure 1a is the IR action spectrum of deprotonated YGGFL, [YGGFL - H]^−^, in the fingerprint region. Notably, the He nanodroplet technique enables resolution of lines in the range of 1640 to 1700 cm^–1^, where three partially resolved lines are attributed to amide I modes (a_6_–a_8_), and one line is assigned to the carboxylate asymmetric stretch (a_5_). Four amide I bands are expected, one for each amide bond in the peptide, but the shoulder visible for line a_6_ and the broadening of line a_7_ may indicate the population of multiple conformers. Lines attributable to the amide II modes (a_2_–a_4_) are also observed, with line a_4_ noticeably shifted with respect to other amide II bands. A diffuse feature, line a_1_, is found in the region of amide III, carboxylate symmetric stretching, and CH bending modes.

**Figure 1:**
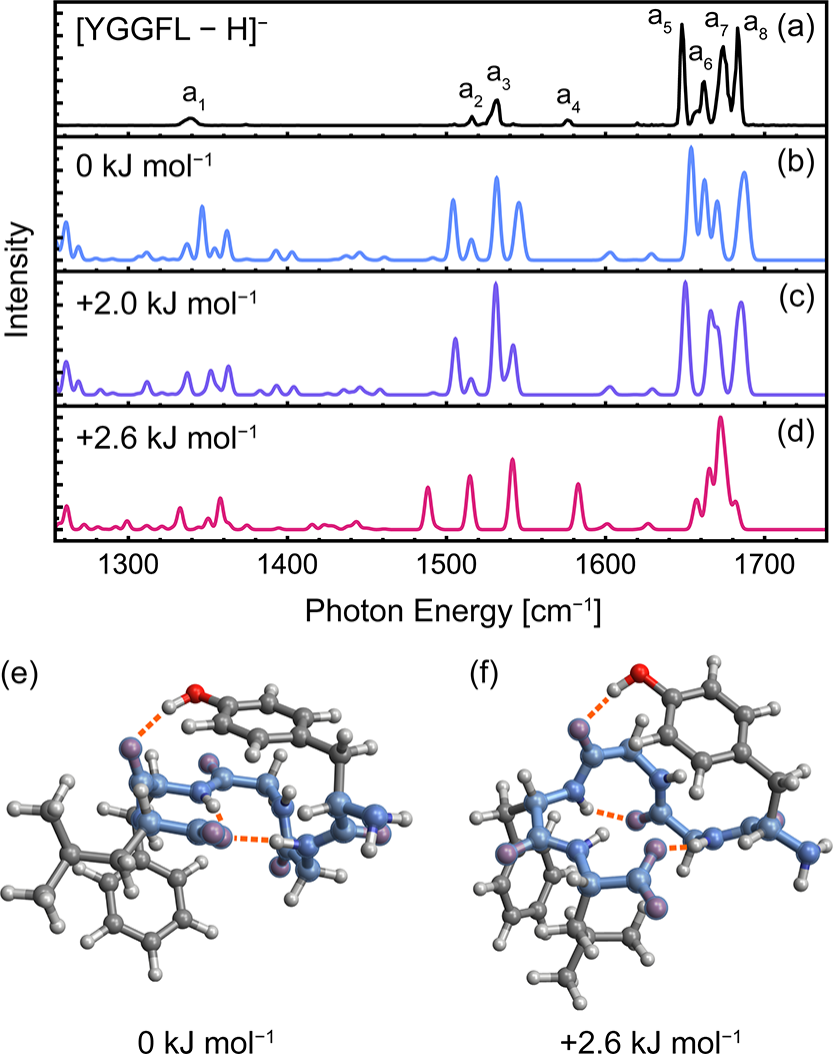
Infrared action spectrum of [YGGFL–H]^−^ captured in helium nanodroplets (a) and computed spectra for the three lowest-energy [Δ­(*E* + *ZPE*)] conformers (b–d) at the CAM-B3LYP­(D3BJ)/def2-TZVP level of theory (0.96 scaling factor). The structure of the lowest- and third-lowest energy conformers are shown in (e) and (f), respectively, with the backbone highlighted in blue. The five lowest-energy structures are shown in Figure S2.

The low-energy conformers of YGGFL were identified by conformational sampling with the GFN2-xtb semiempirical method within CREST and subsequent geometry optimization and vibrational frequency calculation using various density functionals and basis sets. A comparison of basis sets with the B3LYP­(D3BJ) and PBE0­(D3BJ) density functionals showed large changes when progressing from ma-def2-SVP to def2-TZVP but only minor changes between def2-TZVP and def2-TZVPP or ma-def2-TZVP (Figures S6 and S7). Therefore, the def2-TZVP basis set was chosen for further calculations as a balance between accuracy and computational cost. In comparing density functionals, little difference in the relative energy of conformers was observed (Tables S3 and S4). For IR spectra, a Spearman and Pearson coefficient analysis was applied to find the best agreement between experiment and theory and optimize the scaling factors.^97^ The best agreement with the experimental spectrum of [YGGFL - H]^−^ was found using the CAM-B3LYP­(D3BJ)/def2-TZVP level of theory with a scaling factor of 0.96, and this approach was used for all further analysis. A comparison of spectra obtained from various density functionals is shown in Figure S9.

Shown in Figures 1b–d are the spectra of the identified low-energy conformers of [YGGFL - H]^−^ computed at the CAM-B3LYP­(D3BJ)/def2-TZVP level of theory. The lowest-energy conformer (Figure 1e, spectrum Figure 1b) comprises a turn structure featuring two hydrogen bonds to the C-terminal carboxylate and a hydrogen bond between the Tyr side chain and Phe backbone carbonyl (see Figure S5 for a schematic depiction of hydrogen bonds). The second-lowest-energy structure (+2.0 kJ mol^–1^ after zero-point energy (*ZPE*) correction) differs only in the orientation of the Leu side chain (spectrum in Figure 1c). Both structures belong to the family of lowest-energy conformers previously reported by Schinle et al.,^62^ which exhibit a noncanonical turn structure (Ramachandran angles provided in Table S6). The slightly higher-energy conformer shown in Figure 1f exhibits a similar backbone structure with a slightly altered hydrogen bonding pattern. The amide nitrogen of the second glycine residue (Gly_2_) forms a hydrogen bond with an adjacent carbonyl group rather than the C-terminus, and the hydrogen bond of the tyrosine side chain is shifted from the Phe carbonyl to the Gly_2_ carbonyl. In addition, the phenylalanine and leucine side chains are reoriented to facilitate a CH−π interaction. Higher-energy conformers within 10 kJ mol^–1^ of the identified minimum-energy structure were found to have similar backbone configurations and hydrogen bonding patterns to those shown in Figures 1e, f, as illustrated in Figure S2. The lowest-energy phenolate structure was found to be 23.0 kJ mol^–1^ higher in energy (Figure S4, Table S3) and belongs to the family of structures previously reported.^62^ The computed IR spectrum of this deprotomer shows poor agreement with experiment (Figure S10). Therefore, although previous studies of isolated hydroxybenzoic and coumaric acids have demonstrated an energetic preference for the phenolate deprotomer,^102–104^ this property is not observed for the larger YGGFL system.

Good agreement is observed between the experimental spectrum (Figure 1a) and the predicted spectra of the two lowest-energy conformers (Figures 1b, c). The spectrum of the higher-energy conformer (Figure 1d) exhibits poorer overall agreement, but the match with line a_4_, assigned to a blue-shifted amide II mode, is notable. In addition, the blue-shift of the carboxylate asymmetric stretching mode, arising from loss of hydrogen-bonding interactions with the C-terminus, and corresponding shifts of amide I modes may explain the shoulder of line a_6_ and broadening of line a_7_. Thus, the experimental spectrum is consistent with the population of all three conformers. The relative intensity of line a_4_ would suggest a relative population of ca. 10% for the +2.6 kJ mol^–1^ conformer, whereas a Boltzmann population at the trap temperature of 90 K would give a population of ca. 3% (assuming kinetic trapping of conformer population upon capture in He nanodroplets).^98–100^ This discrepancy may arise from the inaccuracy of the applied electronic structure methods or the trapping of population during ion cooling within the trap.^101^ In addition, the nonlinear response inherent to the He nanodroplet method may lead to an incorrect estimation of relative population based on line intensities.^54^

### Spectroscopy and Conformational Analysis of the YGGFL + DIP Complex

Figure 2b displays the IR action spectrum of the complex between deprotonated YGGFL and DIP, [YGGFL + DIP - H]^−^. The spectrum of [YGGFL - H]^−^ is shown in Figure 2a for reference. Upon complexation to DIP, a substantial red-shift of ca. 50 cm^–1^ is observed for the line assigned to the carboxylate asymmetric stretching band (line b_6_ vs line a_5_). In addition, the distribution of lines in the amide I region is compressed, with two closely spaced but broad amide I bands between 1660 and 1690 cm^–1^ (lines b_7_ and b_8_). The carboxylate symmetric stretch (line b_2_) is noticeably blue-shifted from the corresponding line for the unbound species (line a_1_). A new line of moderate intensity is observed at 1276 cm^–1^ (b_1_), in the range of CH and NH bending modes. The strong shift of the lines corresponding to carboxylate symmetric and asymmetric stretching is indicative of the formation of new hydrogen-bonding interactions with the deprotonated C-terminus. Note that DIP introduces new features in the fingerprint region, most notably those arising from the amide modes of this molecule.

**Figure 2:**
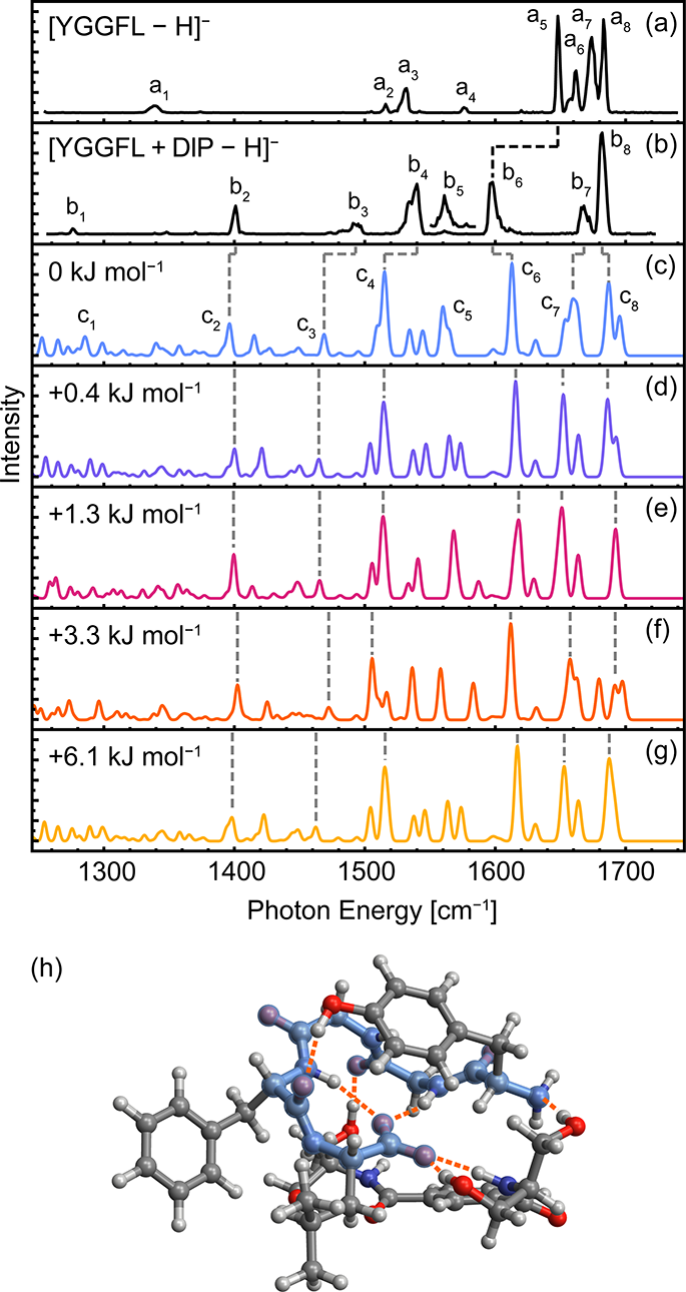
Infrared action spectrum of [YGGFL–H]^−^ (a) and [YGGFL + DIP - H]^−^ (b) captured in helium nanodroplets and computed spectra for the five lowest-energy [Δ­(E+ZPE)] conformers of [YGGFL + DIP - H]^−^ (c–g) at the CAM-B3LYP­(D3BJ)/def2-TZVP level of theory (0.96 scaling factor). The structure of the lowest-energy conformer is shown in (h), with the peptide backbone highlighted in blue. Line b_5_ in (b) is shown with a magnification factor of 10.

Initial conformational sampling of the deprotonated YGGFL+DIP complex was performed with the GFN2-xtb semiempirical method within CREST using default settings. Additional sampling was performed with the LEDE-CREST approach, which is designed to sample the potential energy surface of flexible noncovalent complexes. Structures were subsequently optimized using DFT methods. For the carboxylate deprotomers, similar low-energy structures were found by the two methods, whereas lower-energy phenolate deprotomers were identified by CREST, and lower-energy alkoxide deprotomers were found by LEDE-CREST. All low-energy carboxylate structures (<10 kJ mol^–1^ Δ*E* + *ZPE*, Table S4) were found to adopt a similar peptide backbone configuration, with variation found largely in the orientation of the Leu and Phe side chains and in the hydrogen bonding interactions of the serinol moiety of DIP not coordinated with the carboxylate group, as depicted in Figure S3. Phenolate or DIP deprotomers were found to be substantially higher in energy (+68.4 and +86.2 kJ mol^–1^ Δ*E* + *ZPE*, Table S4).

The lowest-energy complex between YGGFL and DIP (Figure 2h) is characterized by two new hydrogen bonds to the carboxylate moiety as well as two new hydrogen bonds to the peptide backbone, one to the N-terminus and one to the Gly_1_ carbonyl (see Figure S5). Comparing the minimum-energy structure of unbound YGGFL (Figure 1e) to that of the YGGFL + DIP complex (Figure 2h), the hydrogen-bonding configuration of the peptide is preserved, with two hydrogen bonds coordinated to the C-terminus and one hydrogen bond between the Tyr side chain and Phe backbone carbonyl. Overall, the YGGFL structure within the low-energy DIP complex is largely unchanged as compared to the unbound species.

The predicted IR spectra of low-energy conformers of deprotonated [YGGFL + DIP - H]^−^ are displayed in Figures 2c–g, and the structure of the lowest-energy conformer is shown in Figure 2h. Phenolate and alkoxide deprotomers yielded a poor match with the experimental IR spectrum (Figure S11). In comparing the experimental and theoretical IR spectra, discussion here is limited to the minimum-energy structure shown in Figure 2h and corresponding IR spectrum in Figure 2c, as other low-energy conformers adopt a similar structure and exhibit comparable line positions. The amide I bands in Figure 2c are clustered into two groups, which can be divided between hydrogen-bonded amide I (line c_7_) and free amide I (line c_8_) modes. Note that these lines arise from amide I modes of both YGGFL and DIP. This grouping is largely consistent with that observed experimentally (lines b_7_ and b_8_), but the predicted lines are much broader and more widely spaced. Line c_6_, arising from the carboxylate asymmetric stretching mode, is slightly blue-shifted with respect to the corresponding experimental line b_6_. The most intense lines arising from amide II modes, c_4_ and c_5_, also exhibit small shifts in intensity and position from the experimental lines b_4_ and b_5_. Notably, the normal modes associated with these bands involve multiple amide bonds or coupling with side-chain deformation modes, rendering the line positions and intensities sensitive to small structural perturbations (compare Figures 2c–g). Line c_3_ corresponds to CH_2_ scissoring modes and is red-shifted from the assigned experimental line b_3_. Finally, line c_2_ arises from the carboxylate symmetric stretch coupled with CH bending modes, and line c_1_ encompasses several weak CH and NH bending modes.

Agreement between experimental and theoretical IR spectra is notably poorer for the YGGFL + DIP complex than for the unbound peptide. The increased complexity of the noncovalent interactions between YGGFL and DIP may introduce vibrational anharmonicities that partially account for this deviation. However, the agreement between experiment and theory is sufficient to support assignment of the experimental spectrum to the predicted low-energy structure. Although DIP complexation is expected to favor formation of a strongly hydrogen-bonded pocket around the carboxylate moiety,^60^ the structural disruption required to achieve such a binding motif imposes a significant enthalpic penalty. No low-energy structure was identified that featured full coordination of DIP with the C-terminus and favorable intramolecular interactions of YGGFL. Instead, DIP binds to deprotonated YGGFL by alignment below the turn structure, with one serinol moiety coordinating to the carboxylate and one forming a hydrogen bond with a backbone carbonyl. Notably, the low-energy structures of the phenolate and DIP deprotomers do not feature a peptide turn structure, exhibiting instead a pseudolinear peptide arrangement (Figure S4). These arrangements result in significantly reduced backbone hydrogen bonding, contributing to the substantial enthalpic penalty for these deprotomers as compared to the turn-preserving carboxylate structure (+68.4 and +86.2 kJ mol^–1^ Δ*E* + *ZPE*).

### Conformational Sampling of the DIP + Acetate Complex

To better understand the changes in the DIP binding motif imposed by complexation with YGGFL, the conformation of DIP in [YGGFL + DIP - H]^−^ was compared to that in the acetate complex, [CH_3_OO^–^ + DIP]^−^. Note that the low-energy structure of [CH_3_OO^–^ + DIP]^−^ discussed here differs from that previously reported^60^ owing to a more extensive conformational sampling protocol. As shown in Figure 3a, the [CH_3_OO^–^ + DIP]^−^ complex features symmetric intramolecular hydrogen bonds between a hydroxyl group and each amide carbonyl moiety. The acetate anion is located in a binding pocket formed by the remaining four hydrogen bond donors, two serinol OH and two amide NH groups. As shown in Figure 3b, the structural constraints necessary to preserve the turn structure of YGGFL substantially alter this optimized hydrogen bonding network. For [YGGFL + DIP - H]^−^, only one serinol moiety of DIP is coordinated to the carboxylate group, and the orientation of the remaining serinol group is inverted with respect to the aromatic ring to avoid steric clashes with the peptide backbone. This orientation also favors a hydrogen bond to a backbone carbonyl rather than coordination with the carboxylate group. A hydroxyl group on the carboxylate-coordinated serinol also forms a hydrogen bond with the N-terminus rather than an intramolecular hydrogen bond with the amide carbonyl, although this interaction results in only a minor change in the coordination with the charge site. Overall, although the full coordination of the charge site with DIP is favored in the absence of steric constraints, the stability of the turn structure of YGGFL is sufficient to dictate a reduced coordination in the low-energy structure of [YGGFL + DIP - H]^−^. It is notable that the number of hydrogen bonds to the charge site is comparable, although the length and angles of these interactions reduce the overall strength. The flexibility of the DIP hydrogen-bond donors also contributes to the propensity of this reagent to bind with alternate orientations.

**Figure 3:**
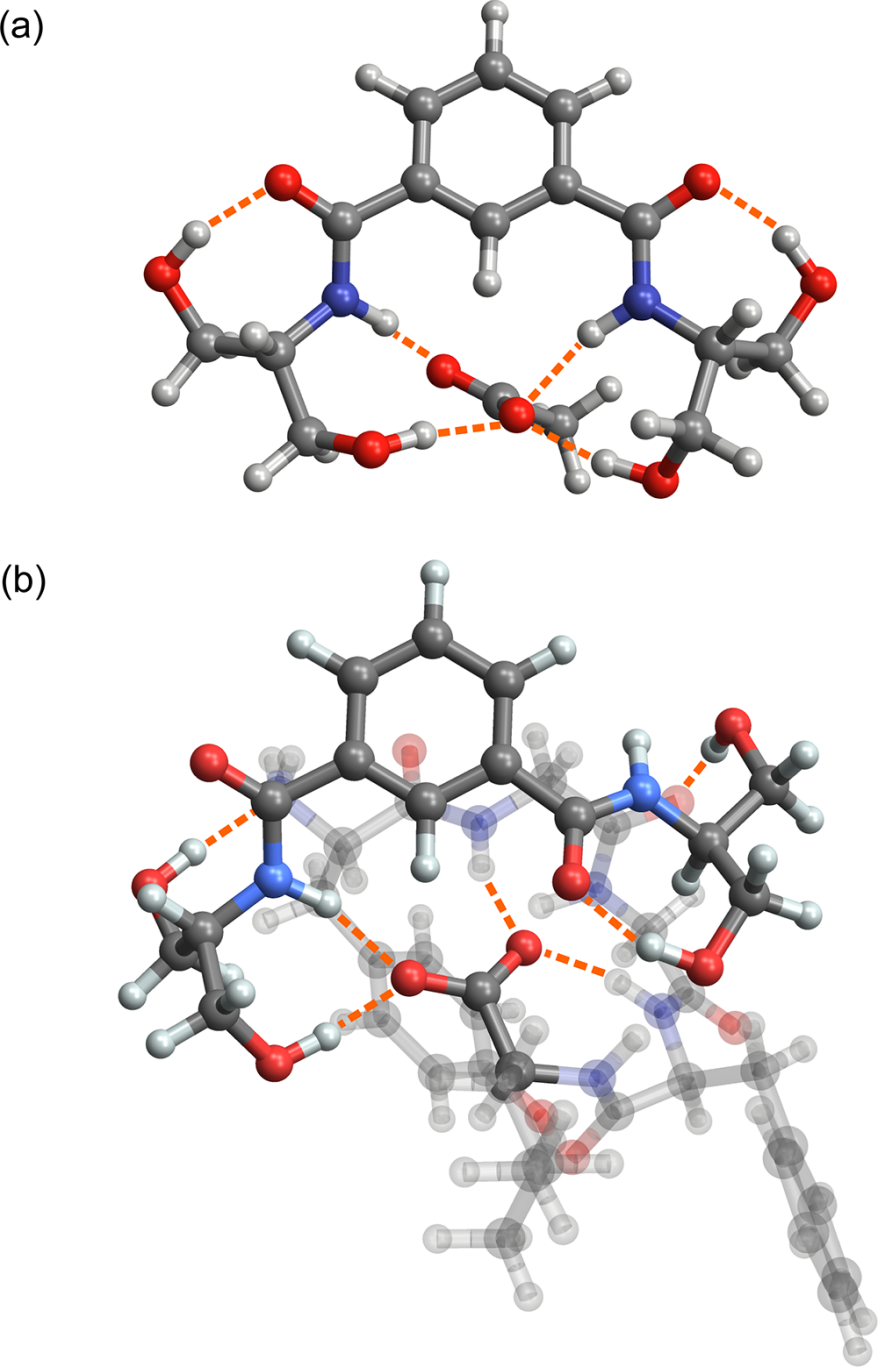
Comparison of the binding motif of the complex between DIP and acetate, [CH_3_OO^–^ + DIP]^−^ (a), and the complex between DIP and deprotonated YGGFL, [YGGFL + DIP - H]^−^ (b). Atoms of YGGFL other than the C-terminus are displayed as translucent for clarity. The structural constraints imposed by interaction with YGGFL substantially alter the observed hydrogen-bonding network with the charge site. Structures were optimized at the CAM-B3LYP­(D3BJ)/def2-TZVP level of theory.

## Conclusions

The structure adopted by biomolecules arises from the balance of intermolecular and intramolecular interactions. Herein, we have investigated changes in the molecular structure of a model peptide upon complexation with an anion-binding reagent. Using cryogenic IR action spectroscopy, we have confirmed the noncanonical turn structure of deprotonated YGGFL, supported by both backbone hydrogen bonding and ionic hydrogen bonding to the C-terminal carboxylate. Perhaps surprisingly, it was found that this structure was largely preserved upon binding of DIP to the peptide, despite the preference of this reagent to encompass carboxylate residues. This result points to the unique stability of the observed turn structure, which is not easily replicated by alternate conformations that could better facilitate DIP coordination at the C-terminus. A similar result was obtained previously for the complex between protonated YGGFL and 18-crown-6, wherein charge site hydrogen bonding was more extensively disrupted while preserving the general backbone conformation.^48^ In addition, the preservation of structure upon capping of a C-terminal hydrogen bond donor in protonated YGGFL was also reported.^47^ Together, these results point to the robustness of peptide backbone structure against perturbations at the chain termini.

## Supplementary Material





## Notes

Schematic of instrumentation, depiction of low-energy conformers, basis set dependence of vibrational frequency calculations, vibrational spectra of low-energy conformers, tabulation of experimental lines, relative energies of electronic structure methods, results of CREST sampling, peptide Ramachandran angles (PDF)
XYZ coordinates from electronic structure calculations, experimental IR action spectra (TXT)

## Abbreviations

DIP: diserinol isophthalamide
ESI-MS: electrospray ionization mass spectrometry
nESI: nano electrospray
FEL: free-electron laser
TOF: time-of-flight
