# Supplementary material for: Examining the Effect of an Anion-Binding Reagent on the Structure of Deprotonated Leucine Enkephalin Using Cryogenic-Ion Infrared Action Spectroscopy
Source: J Phys Chem A. 2025 Aug 28;129(36):8303–11. doi: 10.1021/acs.jpca.5c03984 (PMC12434663; doi:10.1021/acs.jpca.5c03984)
Supplement: Supplementary file 1 [file jp5c03984_si_001.pdf]

Examining the Effect of an Anion-Binding Reagent on the Structure of Deprotonated Leucine Enkephalin Using Cryogenic-Ion Infrared Action Spectroscopy

Madeline Schultz,† Nwanne D. Banor,† Katja Ober,‡ America Y. Torres-Boy,‡ Maleesha T. Fernando,† Miyuru M. Wellalage,† Neil A. Ellis,† Gert von Helden,‡ Daniel A. Thomas†,*

†*Department of Chemistry, University of Rhode Island, Kingston, RI 02881*
‡*Fritz-Haber-Institut der Max-Planck-Gesellschaft, Faradayweg 4-6, 14195 Berlin, Germany*

Table of Contents

Schematic of cryogenic-ion infrared action spectroscopy instrumentation S2

Conformers of [YGGFL – H]⁻ and [YGGFL + DIP – H]⁻ S3

Hydrogen bond map of [YGGFL – H]⁻ and [YGGFL + DIP – H]⁻ S5

Vibrational spectra for lowest-energy YGGFL conformer using PBE0(D3BJ) with different basis sets S6

Vibrational spectra for lowest-energy YGGFL conformer using B3LYP(D3BJ) with different basis sets S6

Vibrational spectra for 5 lowest-energy conformers of [YGGFL – H]⁻ at CAM-B3LYP(D3BJ)/def2-TZVP S6

Vibrational spectra for lowest energy conformer of [YGGFL – H]⁻ with different functionals S7

Vibrational spectra of deprotomers of [YGGFL – H]⁻ and [YGGFL + DIP – H]⁻ S7

Experimental Spectral Lines Measured S8

Relative Energies Of [YGGFL – H]⁻ with Different Functionals S9

Relative Energies Of [YGGFL + DIP – H]⁻ with Different Functionals S9

Conformers Obtained from CREST Sampling S10

Ramachandran angles of minimum-energy conformers of [YGGFL – H]⁻ and [YGGFL + DIP – H]⁻ S10

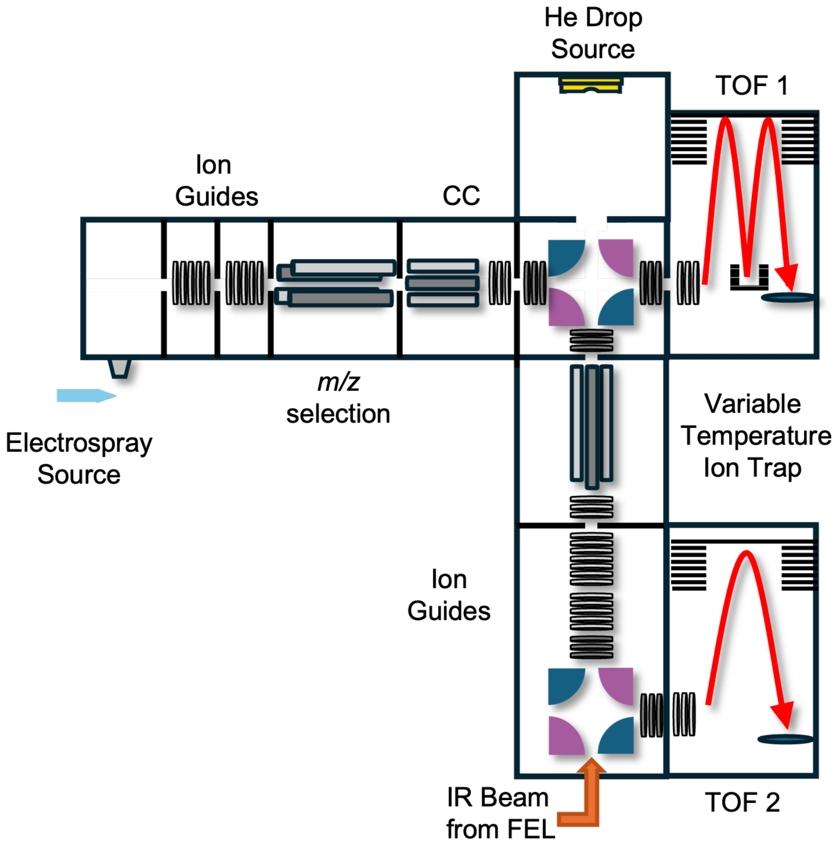

Figure S1. Instrumentation to collect cryogenic ion infrared action spectroscopy measurements at the Fritz Haber Institute of Berlin. Ions generated by a home-built ESI source are mass-selected by a quadrupole mass filter and subsequently bent 90° by a quadrupole deflector to be stored in a variable-temperature ion trap. A pulsed beam of helium nanodroplets traverses the trap, resulting in ion capture. Droplet-entrained ions then travel to ion guides, wherein they are irradiated with infrared photons from the free-electron laser at the Fritz Haber Institute. The successive absorption of multiple photons results in ion release from the helium nanodroplets. Bare ions are then directed to the second time-of-flight (TOF 2) for detection. Measuring ion signal as a function of incident photon energy yields an infrared spectrum.

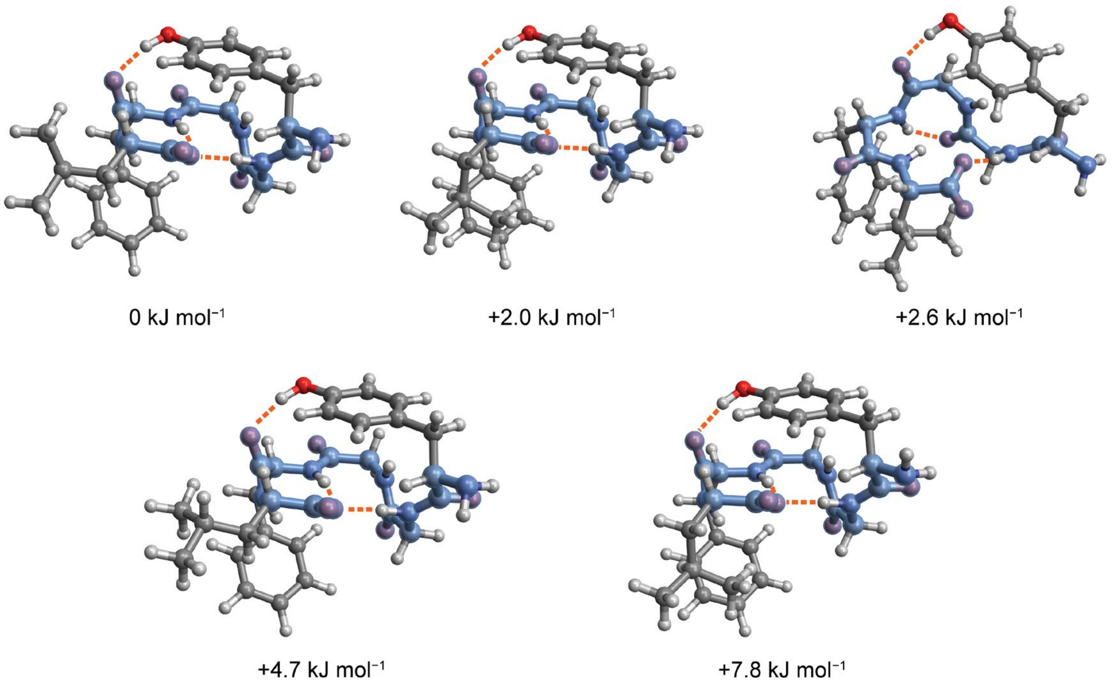

Figure S2. Computed low-energy structures of deprotonated YGGFL, [YGGFL − H]$^-$. Conformers were identified by conformational sampling in the CREST software package and were subsequently optimized at the CAM-B3LYP(D3BJ)/def2-TZVP level of theory. Most structures share a very similar backbone configuration, with the exception of the +2.6 kJ mol^{-1} conformer, which has a distinct hydrogen-bonding pattern. Most differences are found in the orientation of the Phe and Leu side chains.

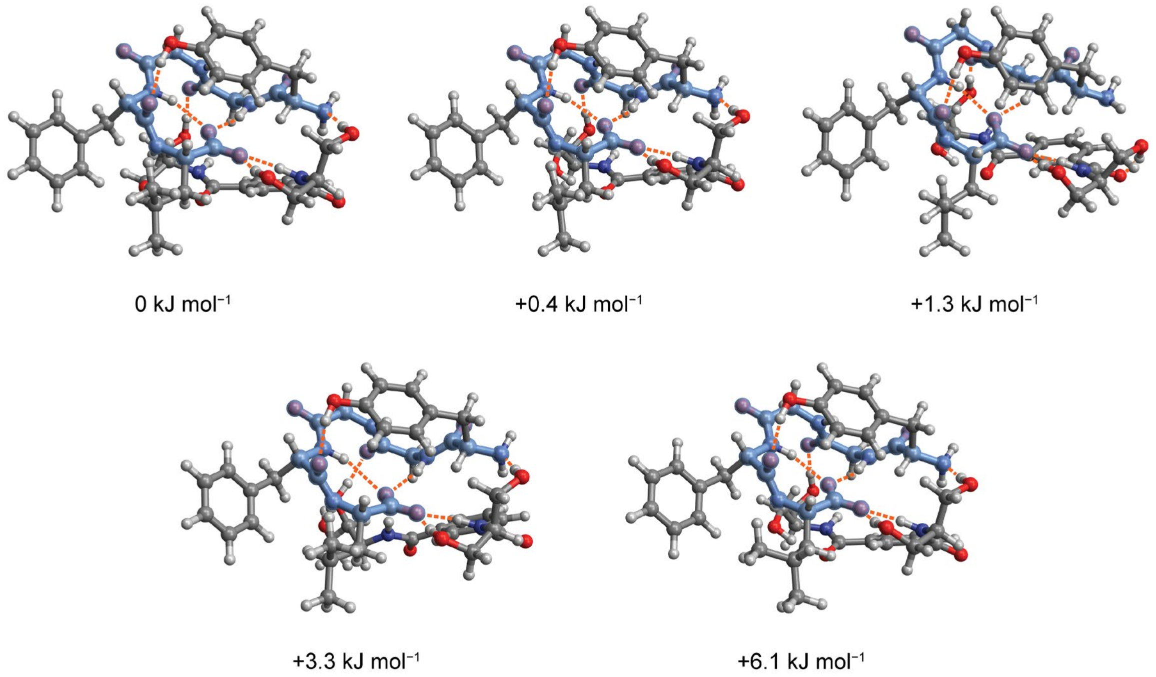

Figure S3. Computed low-energy structures of the complex of deprotonated YGGFL with DIP, [YGGFL + DIP − H]$^-$. Conformers were identified by conformational sampling in the CREST software package and were subsequently optimized at the CAM-B3LYP(D3BJ)/def2-TZVP level of theory. Structures share a very similar backbone configuration, with most differences found in the orientation and hydrogen bonding of the DIP molecule.

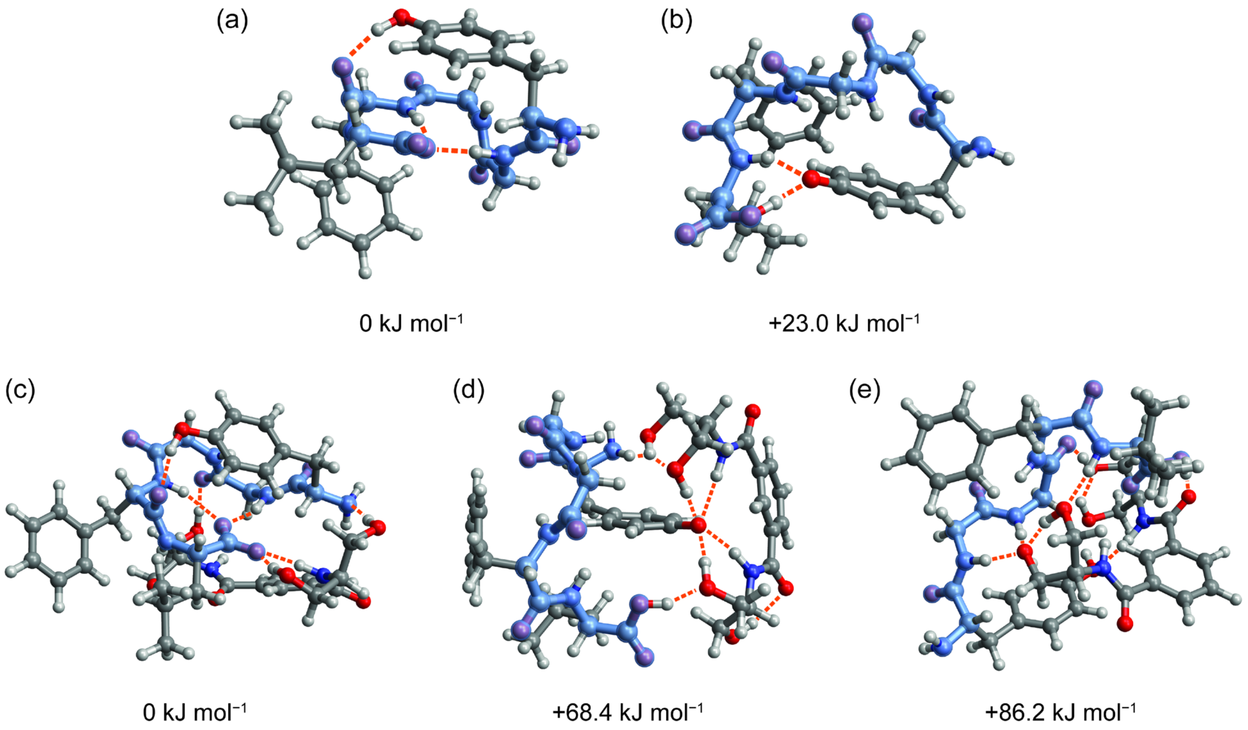

Figure S4. Comparison of computed low-energy structures for deprotomers of YGGFL and YGGFL+DIP. The carboxylate and phenolate deprotomers of $[YGGFL - H]^{-}$ are shown in (a) and (b), respectively. The carboxylate, phenolate, and DIP deprotomers of $[YGGFL + DIP - H]^{-}$ are shown in (c), (d), and (e), respectively. Conformers were identified by conformational sampling in the CREST software package and were subsequently optimized at the CAM-B3LYP(D3BJ)/def2-TZVP level of theory.

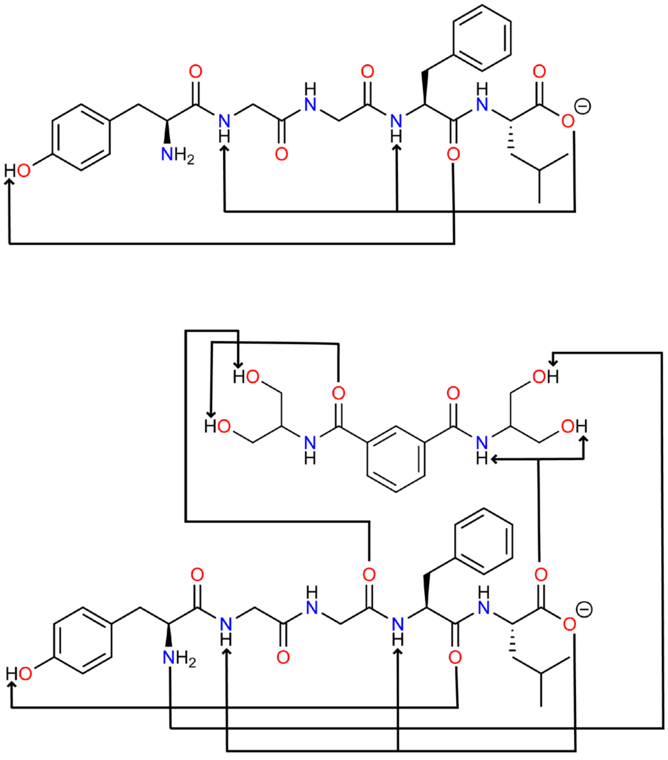

Figure S5. Schematic depiction of the hydrogen-bonding interactions in the low-energy structures of [YGGFL − H]$^-$ (top) and [YGGFL + DIP − H]$^-$ (bottom). All hydrogen bonding interactions of [YGGFL − H]$^-$ are preserved in [YGGFL + DIP − H]$^-$.

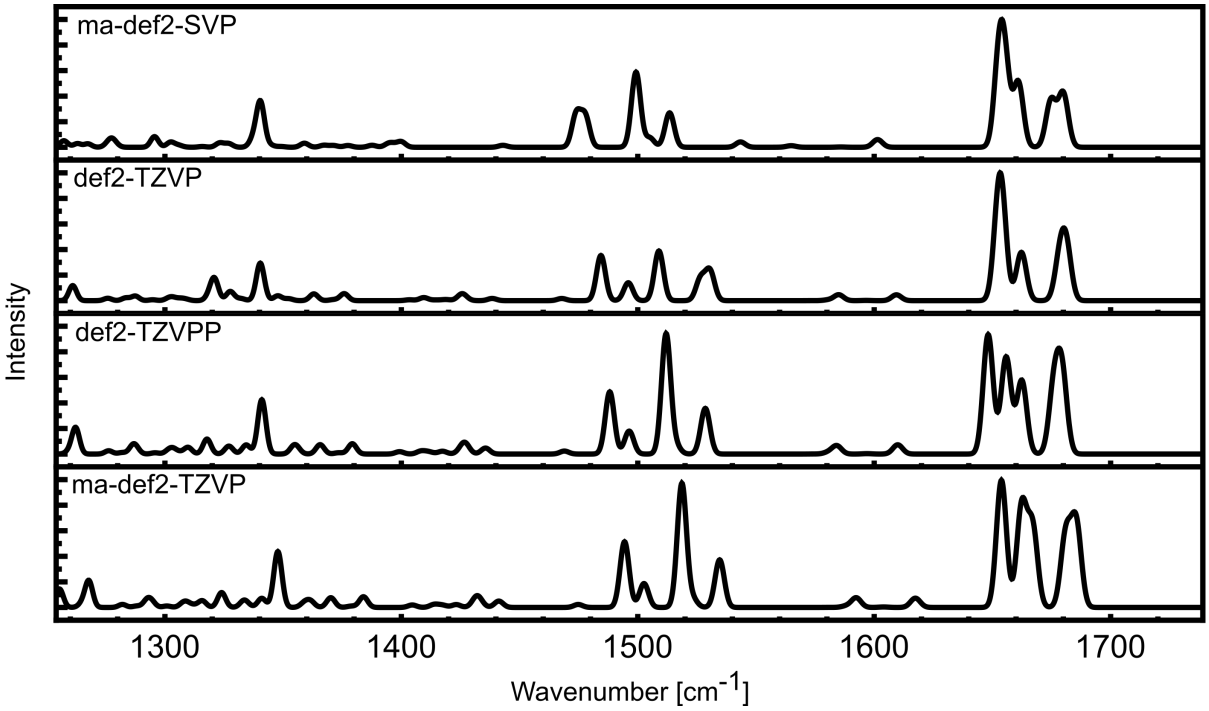

Figure S6. Computed vibrational spectra for lowest-energy conformer of YGGFL anion calculated at PBE0(D3BJ) level of theory with varied basis sets and scaled by best fit to experimental spectra.

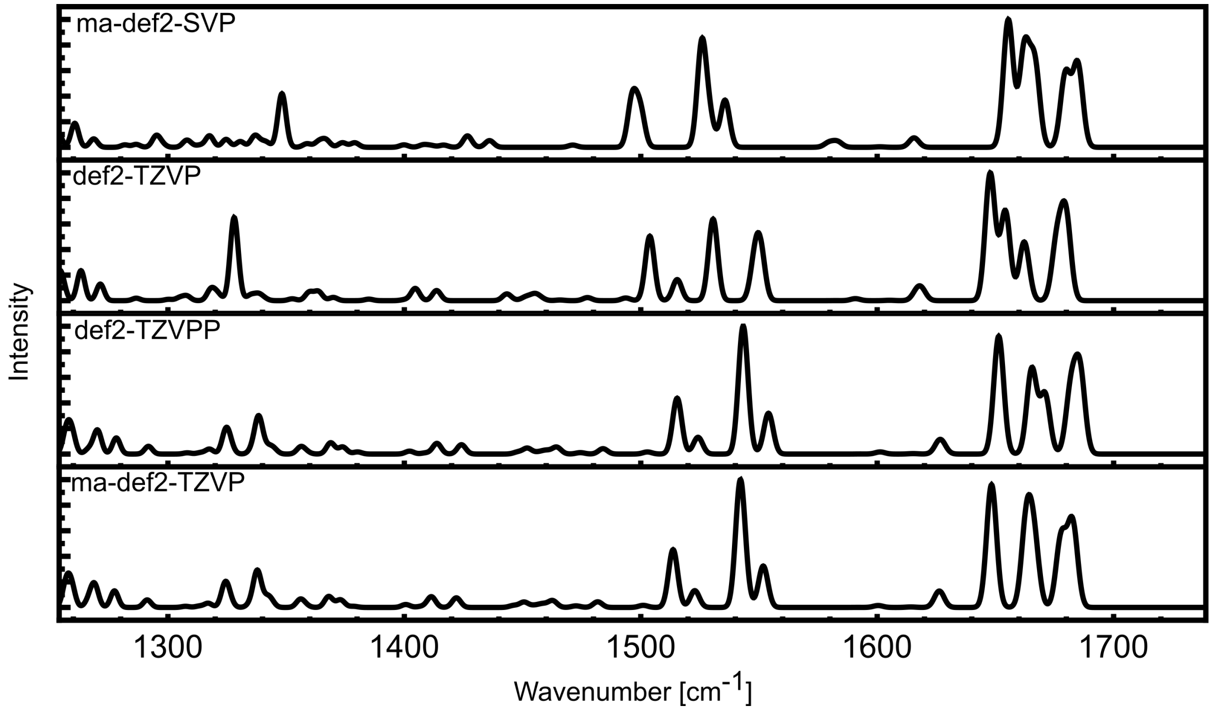

Figure S7. Computed vibrational spectra for lowest-energy conformer of YGGFL anion calculated at B3LYP(D3BJ) level of theory with varied basis sets and scaled by best fit to experimental spectra.

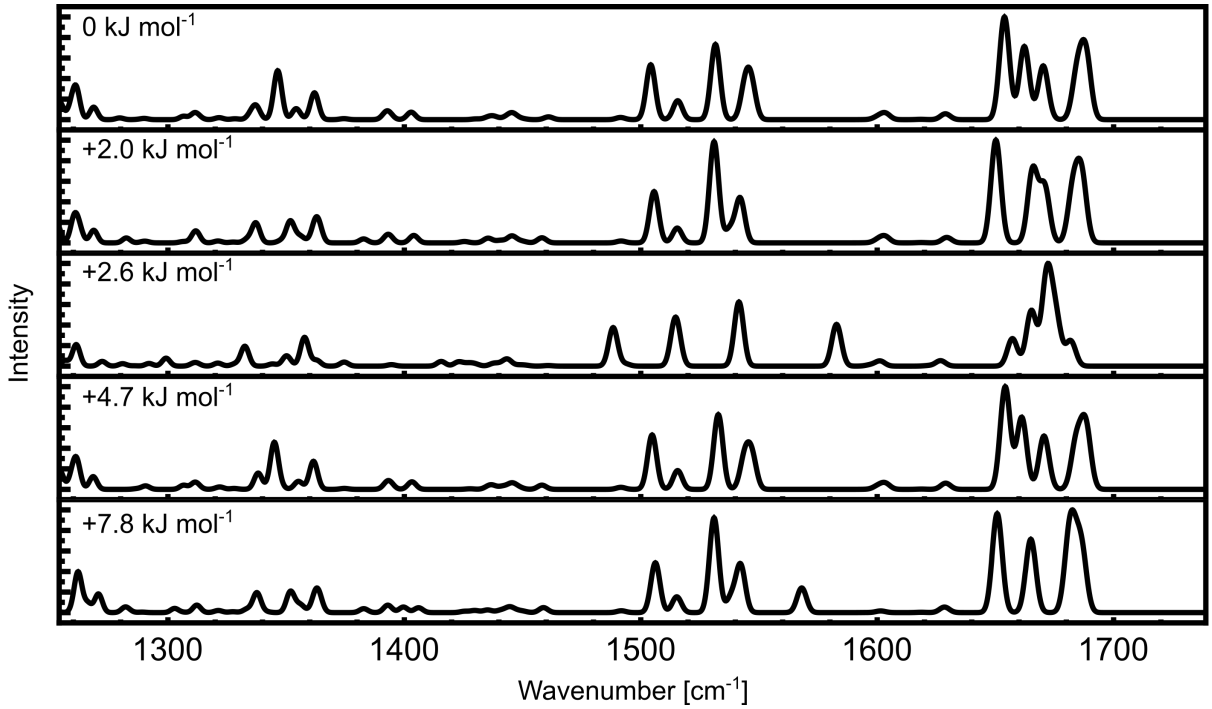

Figure S8. Computed vibrational spectra for the 5 distinct lowest-energy conformations of the YGGFL anion calculated at CAM-B3LYP(D3BJ)/def2-TZVP with a scaling factor of 0.96.

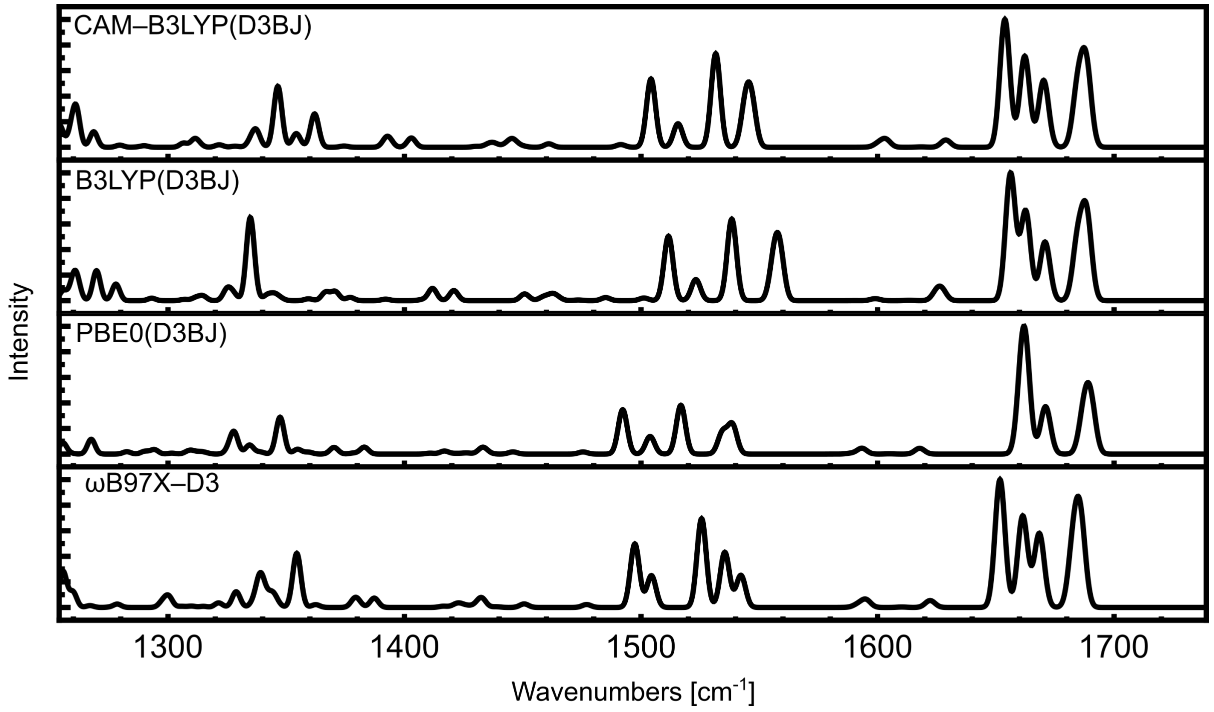

Figure S9. Computed vibrational spectra for the lowest-energy conformer of the YGGFL anion calculated with different density functionals using a def2-TZVP basis set and scaled by best fit to experimental spectra.

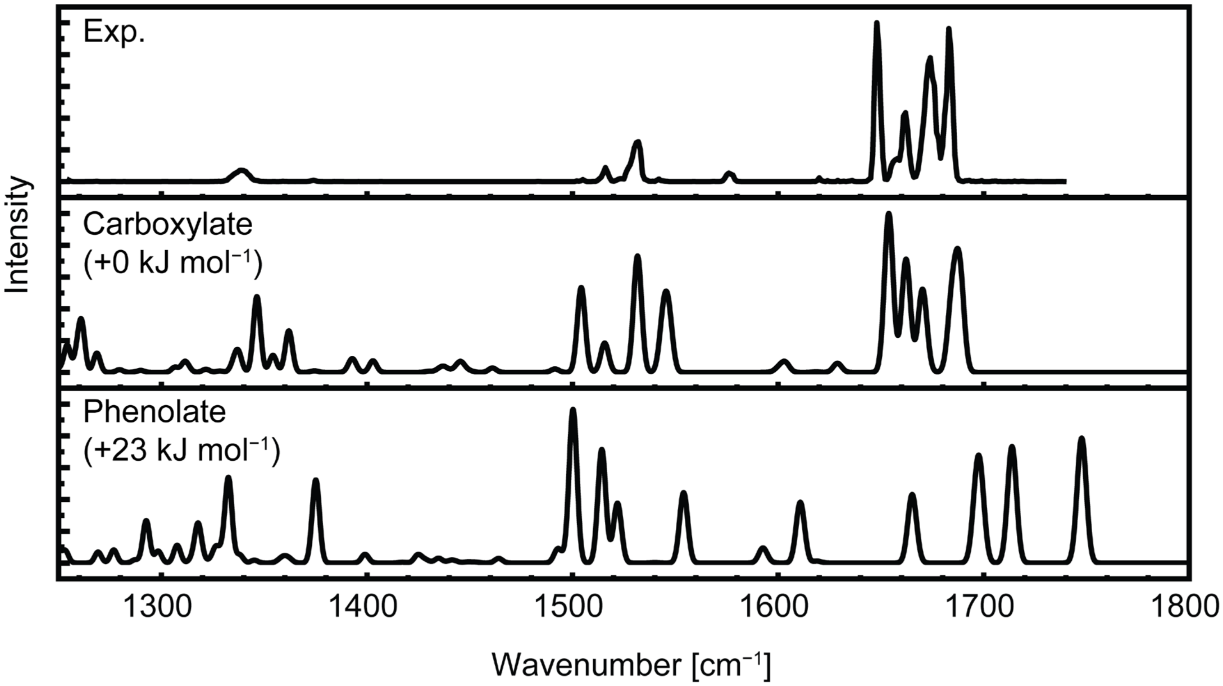

Figure S10. Comparison of experimental and computed vibrational spectra for the lowest-energy conformer of the YGGFL anion (+0 kJ/mol) and lowest-energy phenolate structure (+23 kJ/mol) calculated at the CAM-B3LYP/def2-TZVP level of theory and scaled by 0.96.

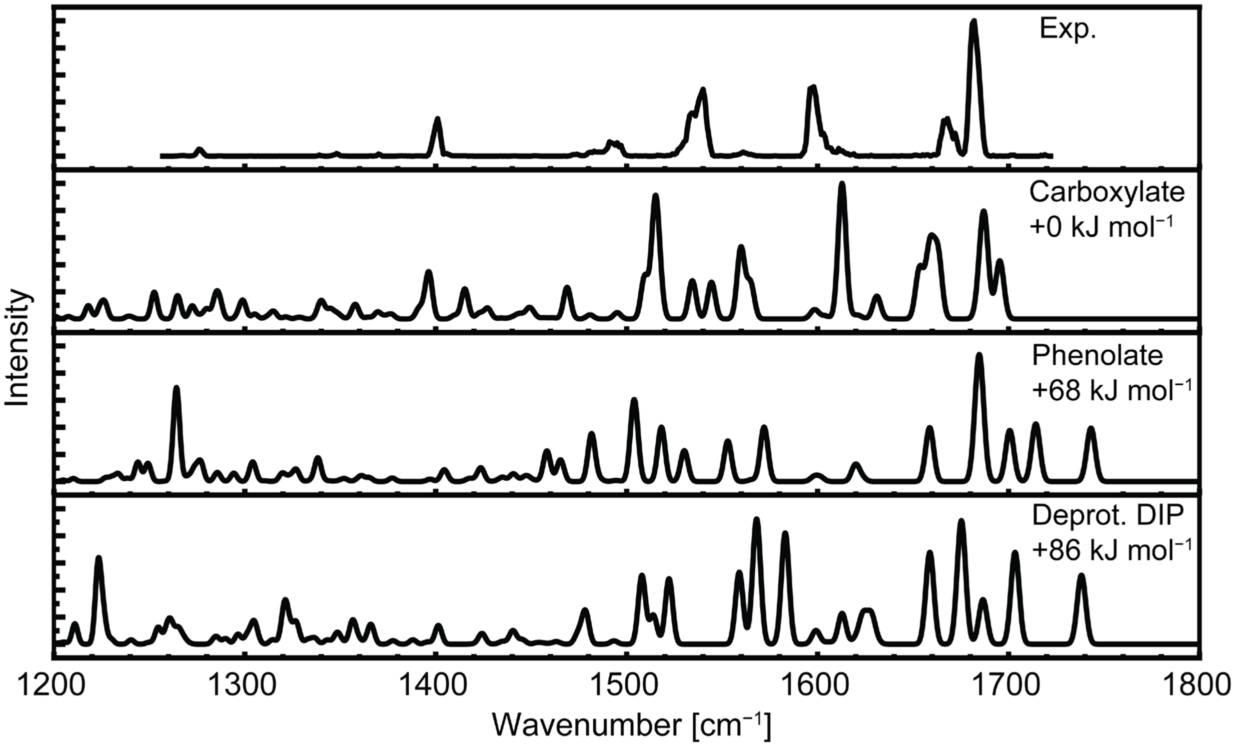

Figure S11. Comparison of experimental and computed vibrational spectra for the lowest-energy conformer of the YGGFL+DIP anion (+0 kJ/mol) and lowest-energy phenolate structure (+68 kJ/mol) and alkoxide structure (+86 kJ/mol) calculated at the CAM-B3LYP/def2-TZVP level of theory and scaled by 0.96.

Table S1. Experimental IR spectral lines measured for $[YGGFL - H]^{-}$.

**Line**	**Wavenumber [cm^{-1}]**
a_1	1339
a_2	1516
a_3	1531
a_4	1577
a_5	1648
a_6	1662
a_7	1674
a_8	1683

Table S2. Experimental IR spectral lines measured for [YGGFL + DIP – H]⁻.

Line	Wavenumber [cm^{-1}]
b_1	1276
b_2	1401
b_3	1493
b_4	1540
b_5	1561
b_6	1597
b_7	1667
b_8	1682

Table S3. Zero-point-corrected relative energies of [YGGFL – H]⁻ conformers with different density functionals calculated with the def2-TZVP basis set.

	**Zero-Point Corrected Relative Energy, Δ(E+ZPE) [kJ/mol]**			
**Conformer**	**B3LYP(D3BJ)**	**CAM-B3LYP(D3BJ)**	**PBE0(D3BJ)**	**ωB97X-D3**
1	0.00	0.00	0.00	0.00
2	1.29	2.02	2.36	0.46
3	4.49	2.58	2.57	3.10
4	4.54	4.69	4.85	4.70
5	7.15	7.82	8.35	6.48
6 (phenolate)	19.96	23.01	16.72	21.12

Table S4. Zero-point-corrected relative energies of [YGGFL + DIP – H]⁻ conformers with different density functionals calculated with the def2-TZVP basis set.

	**Zero-Point Corrected Relative Energy, Δ(E+ZPE) [kJ/mol]**			
**Conformer**	**B3LYP(D3BJ)**	**CAM-B3LYP(D3BJ)**	**PBE0(D3BJ)**	**ωBx97-D3**
1	0.00	0.00	0.00	0.00
2	0.21	0.40	0.18	0.28
3	3.66	1.34	3.73	3.93
4	3.93	3.25	4.12	4.89
5	5.71	6.14	6.09	5.20
6 (phenolate)	-	68.36	-	-
7 (deprotonated DIP)	-	86.22	-	-

Table S5. Conformers Obtained from CREST Conformational Search with varied sampling approaches.

**CREST Method**	**[YGGFL – H]$^-$ (carboxylate)**	**[YGGFL – H]$^-$ (phenolate)**	**[YGGFL + DIP – H]$^-$ (carboxylate)**	**[YGGFL + DIP – H]$^-$ (phenolate)**	**[YGGFL + DIP – H]$^-$ (deprotonated DIP)**	**DIP + CH_3OO^-**
GFN2	314	224	304	397	358	519
nci/GFN2	-	-	95	-	-	-
LEDE-CREST	-	-	187	701	767	-

Table S6. Conformers Obtained from CREST Conformational Search with varied sampling approaches.

**[YGGFL+H]$^+$ [a]**			**[YGGFL−H]$^-$ [b]**			**[YGGFL+DIP−H]$^-$**		
	**φ [°]**	**ψ [°]**		**φ [°]**	**ψ [°]**		**φ [°]**	**ψ [°]**
**Y**	N/A	−62.2	**Y**	N/A	136.4	**Y**	N/A	132.6
**G**	−76.8	78.8	**G**	−111.8	17.3	**G**	−123.1	20.4
**G**	53.4	−132.8	**G**	92.6	4.5	**G**	93.0	5.0
**F**	−61.5	−19.4	**F**	−151.4	−69.5	**F**	−141.6	−79.4
**L**	−70.7	N/A	**L**	−96.6	N/A	**L**	−91.6	N/A

a. Structure as reported in Burke, N. L.; Redwine, J. G.; Dean, J. C.; McLuckey, S. A.; Zwier, T. S. UV and IR spectroscopy of cold protonated leucine enkephalin. *Int. J. Mass Spectrom.* **2015**, *378*, 196-205. DOI: 10.1016/j.ijms.2014.08.012.

b. Structure reported in this work and similar to that first described in Schinle, F.; Jacob, C. R.; Wolk, A. B.; Greisch, J.-F.; Vonderach, M.; Weis, P.; Hampe, O.; Johnson, M. A.; Kappes, M. M. Ion Mobility Spectrometry, Infrared Dissociation Spectroscopy, and ab Initio Computations toward Structural Characterization of the Deprotonated Leucine-Enkephalin Peptide Anion in the Gas Phase. *J. Phys. Chem. A* **2014**, *118* (37), 8453-8463. DOI: 10.1021/jp501772d.
